# Stereospecific Characterization of Triacylglycerols in Vegetable Oils by Chiral Supercritical Fluid Chromatography and Mass Spectrometry

**DOI:** 10.1021/acs.jafc.6c07006

**Published:** 2026-10-24

**Authors:** Marika Kalpio, Dilakshi Naotunna Palliya Guruge, Mikael Fabritius, Qizhu Zhao, Baoru Yang

**Affiliations:** Food Sciences Unit, Department of Life Technologies, 8058 University of Turku, FI-20014 Turku, Finland

**Keywords:** enantiomer, chiral separation, isomeric ratio, supercritical fluid chromatography, triacylglycerol, vegetable oil

## Abstract

Although triacylglycerols (TGs) in vegetable oils are comparatively well characterized at the molecular species level, the detailed stereospecific composition and species-dependent variation remain largely unexplored. This study examined the separation and chiral elution behavior of eight asymmetric TG molecular species using supercritical fluid chromatography mass spectrometry (SFC-MS) with two CHIRALPAK IG-U columns and an acetonitrile/methanol modifier. Following evaluation using TG standards, the chiral SFC-MS method was applied to separate and characterize regioisomers and enantiomers of five asymmetric TG molecular species containing both saturated and unsaturated fatty acyls across 12 vegetable oils. Stereospecific profiles of these TG molecular species varied markedly among plant species, revealing nonrandom patterns and interspecies differences in fatty acyl distribution within the glycerol backbone of TGs. These findings contribute to understanding the chirality in TG biosynthesis in plants and offer valuable information to support targeted food formulation and product development.

## Graphical abstract

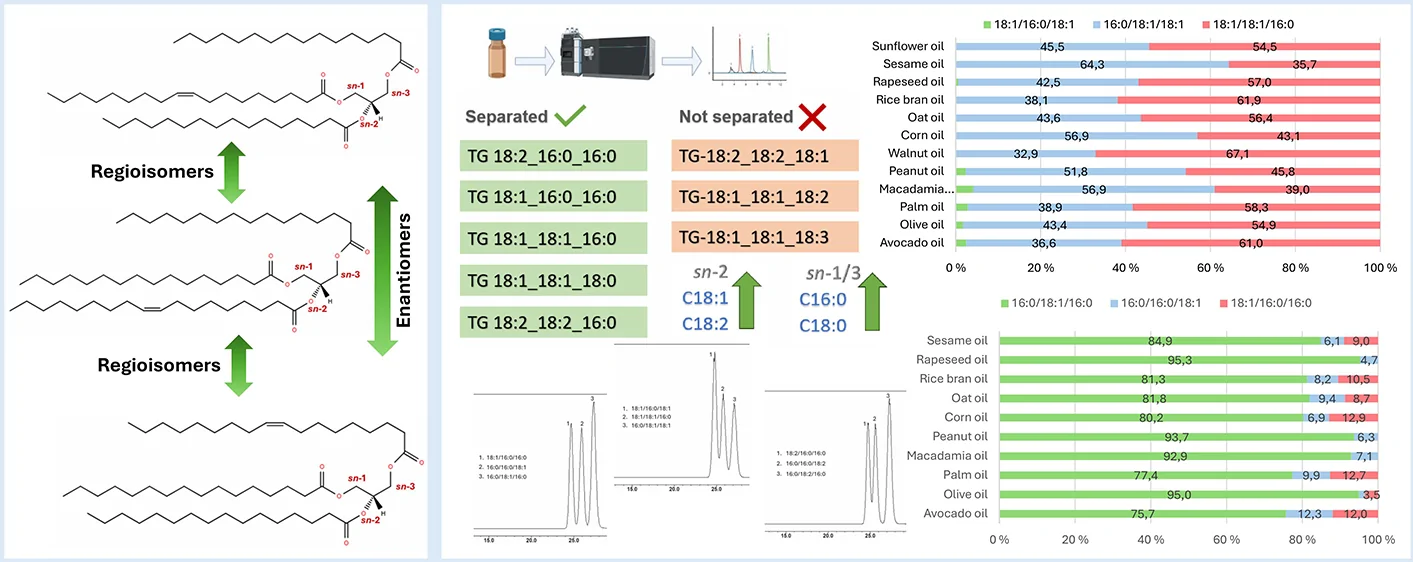

## Introduction

Vegetable oils (VOs) are an essential element of a healthy diet and key raw materials for food, fuel, and various industrial products.^1,2^ They are complex mixtures of lipids, composed primarily of triacylglycerols (TGs), which typically account for 95–98% of total lipids. The composition and distribution of fatty acyls (FAs) at the various stereochemical positions (*sn*-1, 2, and 3) on the glycerol backbone lead to the formation of diverse TG molecular species, as well as enantiomers and regioisomers within TG molecular species.

In the human body, TG isomers can have distinct metabolic outcomes due to the mechanisms of enantiospecific actions of enzymes involved in lipid digestion and metabolism.^3–6^ In food processing, the specific positioning of FAs in TGs can influence physical properties such as melting behavior, which in turn affects flavor, texture, mouthfeel, and cooking characteristics.^3,7,8^ Research has shown that TGs in seeds and other storage tissues serve additional roles besides energy storage,^9^ such as in plant development and stress tolerance.

Most TGs in VOs are composed of a limited number of FAs, including saturated (S) palmitic (C16:0) and stearic acids (C18:0) as well as unsaturated (U) FAs, oleic (C18:1), linoleic (C18:2), and α-linolenic acids (C18:3).^10^ Consequently, a dominating share of the TG abundance in VOs is represented by a few TG molecular species such as 16:0_16:0_18:2, 16:0_18:2_18:2, 16:0_18:1_18:1, 18:2_18:2_18:2, 18:1_18:2_18:2, 18:1_18:1_18:2, 18:0_18:1_18:1, and 18:1_18:1_18:1^10^. Only two of these molecular species are fully symmetric (TG 18:2_18:2_18:2 and 18:1_18:1_18:1), and the others contain an asymmetric carbon and can exist in different isomeric forms.

The TG composition and the positional distribution of FAs in VOs are primarily determined by the genetic control of lipid biosynthetic pathways. These pathways involve acyltransferase enzymes such as glycerol-3-phosphate acyltransferase (GPAT), lysophosphatidic acid acyltransferase (LPAAT), diacylglycerol acyltransferase (DGAT), and phospholipid:diacylglycerol acyltransferase (PDAT), which have different substrate specificities and positional selectivities.^11^ Consequently, the isomeric profiles of TGs are highly species-specific and can vary significantly even within species e.g., among subspecies, varieties, and cultivars, reflecting both genetic diversity and enzymatic regulation.^12–14^ In VOs, monounsaturated FAs (MUFAs), particularly C18:1, exhibit a preference for the *sn*-2 position of TG molecules. SFAs are preferred at the primary positions (*sn*-1/3) of TGs in the majority of VOs.^15–17^

The regioisomeric composition of selected TG molecular species has been investigated in several common VOs such as olive oil, palm oil, and sunflower oil.^15,16,18^ In contrast, studies on TG enantiomers have largely focused on chromatographic separation of reference compounds, and their distribution in natural fats and oils has been explored only rarely due to analytical challenges and the structural complexity of TG molecular species.^19–25^ Chiral-phase chromatography is currently the only approach capable of resolving both enantiomers of TGs. Moreover, simultaneous separation of TG enantiomers and regioisomers has only been successfully demonstrated for a limited number of TG molecular species using specific chiral columns under optimized conditions. Lísa and Holčapek in 2013^19^ demonstrated such separation for several TG molecular species, although coelution limited identification and isomeric ratio determination in some cases. In 2019, Nagai et al. achieved the separation of both enantiomers and regioisomers of TGs 16:0_16:0_18:1, 16:0_18:1_18:1, 16:0_16:0_18:2, 16:0_18:2_18:2, 18:0_18:0_18:1, 18:0_18:1_18:1, 18:0_18:0_18:2, and 18:0_18:2_18:2 without sample recycling.^26^ Later, they reported quantification of regioisomers and enantiomers of TG 16:0_18:1_18:1 and 16:0_16:0_18:1 in palm oil and lard.^27^ Recently, Nilsson and others achieved baseline separation using the same CHIRALPAK IF-3 columns that were used by Nagai et al. after a systematic investigation of the effects of mobile phase composition, temperature, flow rate, and columns coupled in series.^28^ However, high-performance liquid chromatography (HPLC) methods often require long analysis time, consume large amount of organic solvent, and generate large amount of waste to be properly handled.

Supercritical fluid chromatography (SFC) is an attractive alternative due to its reduced solvent consumption, high separation efficiency, compatibility with MS detection without sample recycling, and suitability for nonpolar compounds such as lipids.^29,30^ Various SFC methods have been developed and validated for analyzing a wide range of lipids and their derivatives in different sample types.^31–34^ However, reports on the stereospecific analysis of TGs using SFC remain limited, with only a few studies having been conducted on the separation of TG isomers using SFC.^35–37^ To date, the study by Masuda et al. in 2021 ^35^ is the only one to report the simultaneous separation and quantification of TG enantiomers and regioisomers in VOs using SFC-based methodology. They separated and quantified the enantiomers and regioisomers of TG 16:0_18:1_18:1 in both olive and palm oils.

Recently, our research group characterized the regioisomeric profiles of major TG species in 18 VOs.^17^ In the present study, simultaneous separation and characterization of enantiomers and regioisomers of several SUU- and SSU-type TG molecular species were achieved for 12 VOs using a chiral-phase SFC-MS method adapted from previously published work by Masuda and others.^35^ The novel findings on the stereospecific composition of TGs in VOs provide in-depth insights into the compositional diversity and potential nutritional or functional implications of VOs.

## Materials and Methods

### Nomenclature

The nomenclature applied in this article follows the LIPID MAPS guidelines.^38^ TG molecular species without a defined positional distribution are presented as TG A_A_B in an increasing order of the number of carbon atoms and double bonds (DBs) in the FA chain (e.g., TG 16:0_16:0_18:1). If only one of the *sn*-positions in the TG molecule is specified, regioisomers are denoted with the defined position indicated in parentheses (e.g., TG 16:0_18:1­(*sn*-2)_16:0). Enantiomeric TGs, in which all three FA positions are stereospecifically defined, are expressed in the order *sn*-1/*sn*-2/*sn*-3 (e.g., TG 16:0/16:0/18:1).

### Sample Materials

The VOs avocado (*Persea americana*, Lauraceae), olive (*Olea europaea*, Oleaceae), macadamia nut (*Macadamia integrifolia*, Proteaceae), peanut (*Arachis hypogaea*, Fabaceae), walnut (*Juglans regia*, Juglandaceae), corn (*Zea mays*, Poaceae), oat (*Avena sativa*, Poaceae), rapeseed (*Brassica napus*, Brassicaceae), sesame (*Sesamum indicum*, Pedaliaceae), and sunflower (*Helianthus annuus*, Asteraceae) oils were purchased from the local markets (Turku, Finland). Rice bran oil (*Oryza sativa*, Poaceae) was purchased from Limepop Naturals (Helsinki, Finland), and palm oil (*Elaeis guineensis*, Arecaceae) was obtained from Anton’s Best (Helsinki, Finland).

### Chemicals and Reference Compounds

Ultrapure carbon dioxide (99.9995%) was purchased from Air Products, Allentown, PA. Methanol and acetonitrile (MS grade), and hexane (HPLC grade) were purchased from Honeywell Riedel-de Haën (Seelze, Niedersachsen, Germany). Ammonium formate (≥99.0%) was purchased from Sigma-Aldrich (Saint Louis, MO).

Elution behavior and isomeric separation of eight different TG molecular species found in VOs were studied using the reference compounds listed in Table S1. The racemic reference compounds were purchased from Larodan AB, Solna, Sweden. To aid the determination of the elution order, enantiostructured TGs were synthesized at the Science Institute, University of Iceland, as reported in a previous study.^25^

### Sample Pretreatment

The TGs were solid-phase extracted from the total lipids using Sep-Pak Vac silica 1 cc (100 mg) solid-phase extraction columns (Waters, Dublin, Ireland), as described in the previous study.^17^ In short, the extracted lipid sample, dissolved in 1 mL of diethyl ether, was applied to the conditioned (1 mL of diethyl ether) column. The sample vial was rinsed with 2 mL of diethyl ether, and this wash was also transferred to the column. The TG fraction was subsequently eluted by adding 9 mL of diethyl ether. The solvent was evaporated to dryness under a gentle flow of nitrogen gas at 50 °C. The resulting TGs were then dissolved in 1 mL of hexane and stored at −80 °C until further analysis. The fatty acid compositions of the VO samples were previously determined as fatty acid methyl esters (FAMEs) by gas chromatography-flame ionization detector (GC-FID) and reported in detail.^17^ The same batch of oils was used in the present study.

### Chiral-Phase Supercritical Fluid Chromatographic Analysis

A SFC instrument (Nexera UC, Shimadzu Corporation, Kyoto, Japan) coupled with a triple quadrupole mass spectrometer (LCMS-8045, Shimadzu Corp) with an electrospray ionization (ESI) was used. TGs were separated using two identical CHIRALPAK IG-U columns [amylose tris (3-chloro-5-methylphenyl carbamate), 3.0 mm × 100 mm × 1.6 μm; Daicel Corporation, Japan] connected in series. CO_2_ was used as a mobile phase A and a mixture of acetonitrile and methanol (90:10, v/v) as a modifier. Methanol containing 0.1% ammonium formate (w/v) was used as a makeup solvent with a flow rate of 0.1 mL/min. Initially, the optimal injection concentration was tested using the method described by Masuda et al. in 2021.^35^ The concentration of TG fraction of oil samples was 0.1 mg/mL in hexane, whereas the concentration of reference compounds was 0.01 mg/mL in hexane. The performance of 15 chromatographic settings (Table S2) was compared to optimize the SFC conditions, including back pressure, column oven temperature, flow rate, gradient of modifier, and injection volume.

### Mass Spectrometric Analysis

The triple quadrupole mass spectrometric analysis was performed using the positive ion mode with ESI under the following settings: interface temperature, 300 °C; desolvation temperature, 526 °C; desolvation line temperature, 150 °C; heat block temperature, 400 °C; nebulizer gas flow, 3.0 L/min; drying gas flow, 10 L/min. In full scan mode, *m*/*z* 750–950 was used. For product ion scan experiments, the corresponding ammonium adduct ion, [M+NH_4_]^+^, of each TG species was selected as the precursor ion, and product ions were monitored over the *m*/*z* range 500–700. In multiple reaction monitoring (MRM) mode, the [M+NH_4_]^+^ adducts and the [DG+H]^+^ fragments were chosen as the precursor and the product ions (Table 1).

**Table 1:** *m/z* Values Used with MRM Setup for each TG Species

**TG species**	**ammonium adduct and product ion**
16:0_16:0_18:2	*m*/*z* 848.80 > 551.50
16:0_16:0_18:1	*m*/*z* 850.80 > 551.50
16:0_18:1_18:1	*m*/*z* 876.80 > 603.50
16:0_18:2_18:2	*m*/*z* 872.80 > 599.50
18:1_18:2_18:2	*m*/*z* 898.80 > 599.50
18:1_18:1_18:3	*m*/*z* 898.80 > 599.50
18:1_18:1_18:2	*m*/*z* 900.80 > 603.50
18:0_18:1_18:1	*m*/*z* 904.80 > 603.50

### Data Handling and Isomer Identification Strategy

Data processing was performed using the LabSolutions software (v. 5.118, Shimadzu Corp). Peak resolutions (R_s_) were computed between isomeric TG species to determine the optimal SFC conditions. The chromatographic parameters that obtained the highest R_s_ values were selected as the optimal conditions for isomeric separation. For partially resolved peaks, the calculated R_s_ values should be considered approximate, as peak width determination depends on the integration algorithm used.

$$resolution\;R_{s}=\frac{\left(t_{b}-t_{a}\right)}{1/2\left(w_{b}+w_{a}\right)}$$

The retention times and chiral elution orders of available TG reference compounds (Table S1) were established under the same chromatographic conditions as those used for the analysis of VOs. The reference-compound mixtures used to identify the regioisomers and enantiomers of each TG molecular species are presented in Table 2. Identification was performed in two steps. First, the chromatographic separation of the three isomers within each TG molecular species was evaluated by coinjecting a racemic reference compound, comprising the two asymmetric enantiomers, with the corresponding symmetric regioisomer. Second, the elution order of the two enantiomers was determined by coinjecting the racemic reference compound with an excess of the corresponding enantiostructured TG reference compound. The complete elution order of the symmetric regioisomer and the two enantiomers was established by combining the results of these two coinjection experiments. An exception was TG 18:0_18:1_18:1, for which the stereospecific elution order of the two enantiomers could not be directly confirmed because a corresponding enantiopure reference compound was unavailable.

**Table 2:** TG Reference-Compound Mixtures Used to Identify TG Isomers

TG species	regioisomer identification	enantiomer identification
16:0_16:0_18:2	16:0_16:0(*sn*-2)_18:2: 16:0_18:2(*sn*-2)_16:0 50:50	16:0_16:0(*sn*-2)_18:2: 18:2/16:0/16:0 75:25
16:0_16:0_18:1	16:0_16:0(*sn*-2)_18:1: 16:0_18:1(*sn*-2)_16:0 75:25	16:0_16:0(*sn*-2)_18:1: 16:0_18:1(*sn*-2)_16:0: 18:1/16:0/16:0 37.5:12.5:50
16:0_18:1_18:1	16:0_18:1(*sn*-2)_18:1: 18:1_16:0(*sn*-2)_18:1 60:40	16:0_18:1(*sn*-2)_18:1: 18:1_16:0(*sn*-2)_18:1: 18:1/18:1/16:0 30:20:50
16:0_18:2_18:2	16:0_18:2(*sn*-2)_18:2: 18:2_16:0(*sn*-2)_18:2 50:50	16:0_18:2(*sn*-2)_18:2: 18:2/18:2/16:0 75:25
18:0_18:1_18:1	18:0_18:1(*sn*-2)_18:1: 18:1_18:0(*sn*-2)_18:1 50:50	

VO samples were then analyzed using the same chromatographic and mass spectrometric conditions. Peak identifications were made by comparing retention times and elution orders observed in the samples with those established for reference compounds. Additional confirmation was obtained from extracted ion chromatograms (EIC) of the corresponding ammonium adduct ions, MRM chromatograms, and literature-reported retention behavior under comparable chiral chromatographic conditions.^35,39^ Product-ion spectra were evaluated to further support peak assignments. The relative intensities of the diagnostic DG fragment ions were compared between chromatographic peaks. Enantiomeric pairs consistently exhibit identical DG fragment-ion intensity ratios, whereas symmetric and asymmetric regioisomers show characteristic differences in these ratios.

For two TG species (16:0_16:0_18:1 and 16:0_18:1_18:1), partial chromatographic overlap occurred, causing isotopic interference. In these cases, the monoisotopic ion (M+0) of a target TG overlapped with the M+2 isotope peak of a more unsaturated TG differing by one DB. Specifically, for TG 16:0_16:0_18:1 (50:1, [M+NH_4_]^+^, *m*/*z* 850.8) interference originated from the M+2 isotope of TG 50:2 ([M+NH_4_]^+^, *m*/*z* 848.8), while for TG 16:0_18:1_18:1 (52:2, [M+NH_4_]^+^, *m*/*z* 876.8) interference originated from the M+2 isotope of TG 52:3 ([M+NH_4_]^+^, *m*/*z* 874.8). To address this, an isotope-distribution correction was applied. Isotopic patterns of the interfering TGs were calculated from their elemental compositions using natural isotope abundances, and the expected M+2 contribution was subtracted from the overlapping EIC.^40^ The relative quantities of each TG isomer in VOs were thus obtained from EIC peak areas after corrections when isotopic overlapping was identified. A detailed description of the isotope-correction algorithm with an example is provided in the Supporting Information.

## Results and Discussion

### Method Adaptation and Evaluation

The method described by Masuda et al.^35^ was adopted as a starting point, and the concentration of the TG reference compound (0.01 mg/mL) was selected based on preliminary evaluation of 0.1 μg/mL, 0.01 mg/mL, and 0.1 mg/mL to ensure adequate signal intensity. A set of key chromatographic parameters, including back pressure (10–16 MPa), column oven temperature (25–35 °C), flow rate (0.4–0.8 mL/min), and injection volume (0.5–1.0 μL), was systematically varied to assess their influence on enantiomeric resolution and chromatographic performance (Table S2). TG 18:1_18:1­(*sn*-2)_16:0 dissolved in hexane (0.01 mg/mL) was used as the reference compound for these evaluations. The objective of this work was to evaluate the applicability of the previously reported chiral SFC conditions for stereospecific analysis of TGs in VOs.

Back pressure showed only a minor influence on resolution under the applied gradient conditions (Figure 1A). It is important to notice that under the experimental conditions used in this study, chromatographic behavior was influenced more strongly by modifier composition than by variations in back pressure due to the relatively high proportion of organic modifier in the mobile phase.^41^ Column oven temperature affected both retention and separation, with lower temperatures generally resulting in improved enantiomeric resolution (Figure 1B), consistent with previous observations in SFC separations of TGs.^42^ Flow rate and injection volume exhibited expected effects on chromatographic behavior (Figure 1C–D). Reduced flow rates and smaller injection volumes improved resolution, whereas higher values decreased analysis time but resulted in broader peaks. These effects may also reflect solvent-mobile phase interactions under the applied conditions. Hexane was selected as the sample solvent due to its compatibility with CO_2_-rich mobile phases in SFC, making it suitable for the dissolution of nonpolar TGs. However, under gradient conditions with increasing proportions of organic modifier, solvent-mobile phase interactions may influence chromatographic behavior, particularly at higher injection volumes.

**Figure 1:**
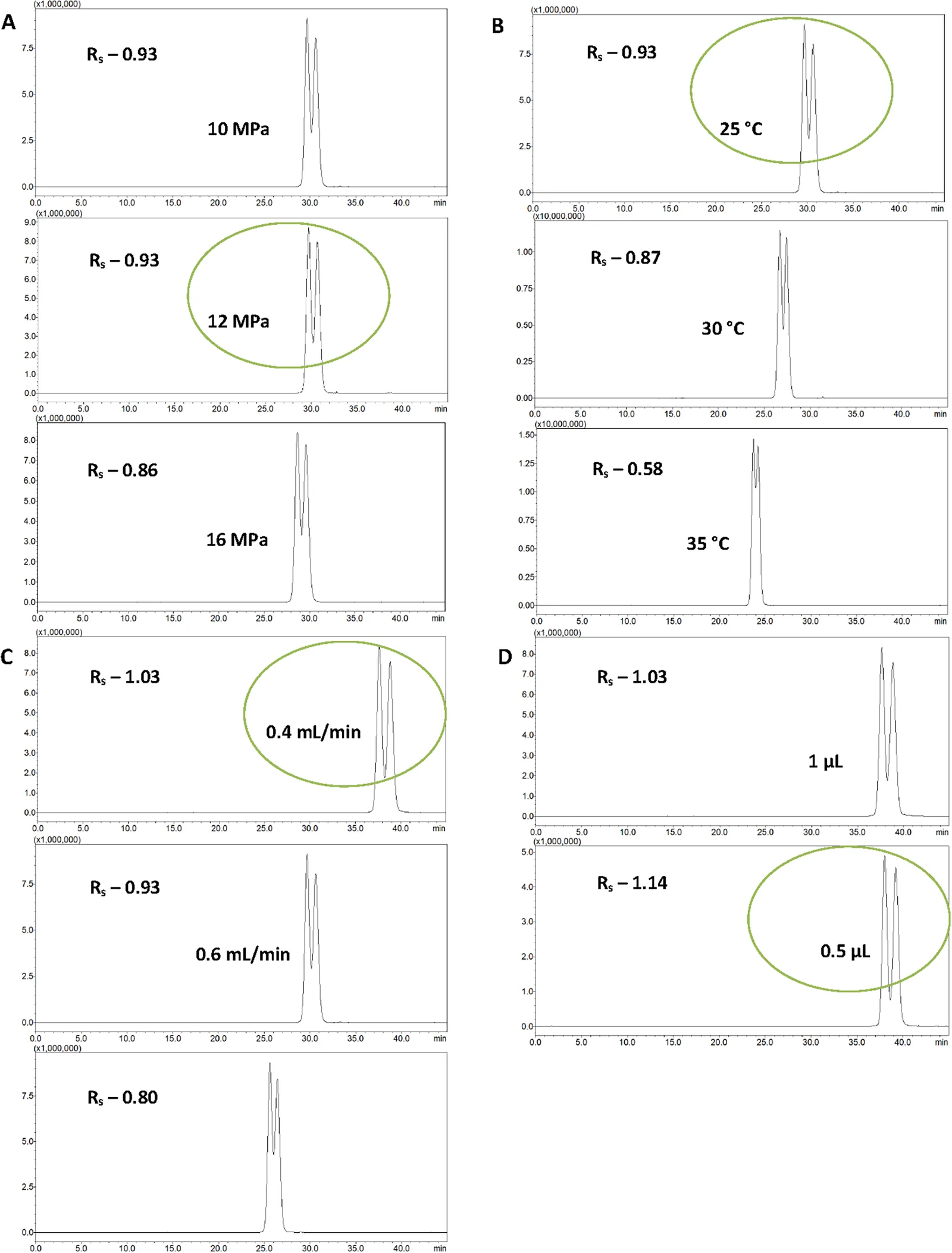
SFC-MS MRM chromatograms showing enantiomeric separation (resolution) of 16:0_18:1­(*sn*-2)_18:1 obtained using different settings (A) back pressures at constant column oven temperature and flow rate (25 °C, 0.6 mL/min); (B) column oven temperatures at constant back pressure and flow rate (10 MPa, 0.6 mL/min); (C) flow rates at constant back pressure and column oven temperatures (10 MPa, 25 °C); (D) injection volumes at constant back pressure, column oven temperature and flow rate (12 MPa, 25 °C, 0.4 mL/min).

In chiral separations of structurally similar lipid molecules, simultaneous optimization of resolution, peak shape, and analysis speed is challenging due to the complex and not fully predictable multiple interactions between TGs and the chiral stationary phase.^43,44^ In particular, the separation of TG enantiomers relies on relatively weak stereoselective interactions, which limit resolution under practical conditions. Therefore, the selected conditions represent a compromise between adequate stereoisomer separation and acceptable analysis time.

Different elution modes were also evaluated (Table S2). Gradient elution provided more effective separation compared to isocratic conditions and was therefore selected for subsequent analyses. The final chromatographic settings (Method No. 15, Table S2) applied were as follows; injection volume, 0.5 μL using the sample loop injection method; flow rate, 0.4 mL/min; modifier gradient: 5–35% (10 min), 35–40% (25 min), 40–5% (0.01 min), and 5% (3 min); column oven temperature, 25 °C; back pressure, 12 MPa. The longer modifier gradient described by Masuda et al.^35^ [modifier gradient 5–35% (50 min), 35–5% (25 min), and 5% (5 min)] was applied to analyze TG isomers of 18:0_18:1_18:1 and 16:0_18:2_18:2 (Figure S7).^35,39^

In the final method subcritical conditions were applied, and most chromatographic conditions were adopted from Masuda et al.^35^ However, the total column length was 50 mm shorter, and the back pressure regulator was set to be 2 MPa higher, the flow rate was reduced from 0.6 mL/min to 0.4 mL/min, and the injection volume was reduced from 1.0 to 0.5 μL compared to the conditions described by Masuda et al. Additionally, the modifier gradient in the present study was increased from 5% to 35% within the first 10 min, followed by an increment from 35 to 40% over the subsequent 25 min. In contrast, Masuda et al. employed a more gradual gradient, increasing the modifier concentration from 5 to 35% over the first 50 min (Figure S7). It should be noted that, in chiral separations of structurally similar TG isomers, achieving simultaneous optimization of resolution, peak shape, and analysis time is inherently challenging because the separation of regioisomers and enantiomers relies on relatively weak stereoselective interactions with the chiral stationary phase. Similar challenges have been addressed through column coupling to increase chromatographic resolving power for TG isomer separations.^28^

### Identification of Triacylglycerol Isomers

The identities and elution orders of the regioisomers and enantiomers of four TG molecular species were determined using a two-step coinjection strategy with reference compounds, whereas the stereospecific elution order of the TG 18:0_18:1_18:1 enantiomers was tentatively assigned (Table 2). In the first step, each racemic reference compound was coinjected with the corresponding symmetric regioisomer. The two enantiomers of the racemic reference compound produced chromatographic peaks with approximately equal areas. The additional peak observed following coinjection was assigned to the symmetric regioisomer (Figures S1A–S4A, S5).

In the second step, the racemic reference compound was coinjected with the excess of one enantiopure structured reference compound. The increase in the area of one peak relative to the other enantiomer peak in the enantiomeric pair identified the added enantiomer, thereby establishing the elution order of the two enantiomers (Figures S1B–S4B). The theoretical proportions of the isomers in the injected mixtures and the identifications are presented in Supporting Information. For TG 18:0_18:1_18:1, for which there was no corresponding asymmetric enantiostructured TG reference compound, the elution order of its individual enantiomers was tentatively assigned based on the analogous elution patterns observed for the other two SUU-type TGs 16:0_18:1_18:1 and 16:0_18:2_18:2, under identical chromatographic conditions. Both reference TG species followed the same elution order, supporting the proposed elution order of 18:0_18:1_18:1.

The established retention times and elution orders were subsequently used to identify TG isomers in VOs. The EICs of the corresponding ammonium adducts, [M + NH_4_]^+^, were evaluated for each TG molecular species. The assignments were further supported by the corresponding MRM chromatograms, product-ion spectra, and, when applicable, retention patterns reported in the literature under comparable chiral chromatographic conditions. Potential contributions from other TG species with the same mass were also evaluated based on the mass-spectrometric data and their previously reported occurrence in VOs.^17^ The identification of TG 16:0_16:0_18:2 illustrates this strategy. First, the retention times and elution order of the peaks detected in the EIC were compared with those established in the coinjection experiments using the corresponding reference compounds (Figure S1). Peaks matching the established retention times were selected for further evaluation. The corresponding MRM chromatograms showed the same chromatographic peak pattern, providing additional support for the selective detection and assignment of the TG molecular species. Product-ion spectra and relative intensities of diagnostic DG fragment ions were then examined to provide additional evidence for distinguishing the symmetric regioisomer from the two asymmetric enantiomers, based on the established differences in the MS/MS fragmentation behavior of TG regioisomers.^45^ The relative intensities of the diagnostic DG fragment ions at *m*/*z* 551.5 and 575.4 were compared among the chromatographic peaks. The two enantiomers, 16:0/16:0/18:2 and 18:2/16:0/16:0, showed comparable relative intensities of the fragment ions, 42:58 (Figure S6B1), as expected because enantiomers exhibit equivalent fragmentation under the achiral mass-spectrometric conditions applied. In contrast, the symmetric regioisomer, 16:0_18:2­(*sn*-2)_16:0, showed a distinctly different fragment-ion intensity ratio of 30:70 (Figure S6A1). Product-ion spectra of the symmetric and asymmetric isomers of TG 16:0_16:0­(*sn*-2)_18:2, 16:0_16:0­(*sn*-2)_18:1, 16:0_18:1­(*sn*-2)_18:1, 16:0_18:2­(*sn*-2)_18:2, and 18:0_18:1­(*sn*-2)_18:1 are provided in Figure S6 in the Supporting Information. VO samples were identified based on the combined chromatographic and mass-spectrometric evidence.

### Chiral Chromatographic Elution Behavior and Isomeric Separation of Triacylglycerols

Despite rapid advances in SFC instrumentation and applications, retention mechanisms for TGs remain less understood compared to HPLC. As is commonly seen with reversed-phase chromatography, a positive correlation is observed between retention time and equivalent carbon number (ECN) when a similar elution gradient is used (Table 3). This has also been observed with chiral liquid chromatographic methods.^26,46^ In this study, this trend was evident, but there were some exceptions. For example, isomers of TG 16:0_16:0_18:2 (ECN 46) had very similar retention behavior with isomers of 16:0_18:1_18:1 (ECN 48). A longer elution gradient was required to separate all three isomers of the TG molecular species 16:0_18:2_18:2, as well as 18:0_18:1_18:1. A slight trend was observed with TG species having more DBs showing weaker retention and eluting earlier. Furthermore, within the same ECN, retention time decreased as the length of the acyl chains and number of DBs increased. Altogether, nine TG isomers containing one or two SFA (C16:0) and one or two UFAs (C18:1 or 18:2) eluted within a narrow retention window of 5.6 min (24.8–30.4 min), indicating that the chiral column and mobile phase did not strongly discriminate between these TG species based on their degree of unsaturation.

**Table 3:** Retention times and Resolutions of Experimented TG Isomers Listed in Order of ECN Values

TG molecular species	ECN	ACN	DB	TG isomer	retention time (min)	resolution (*R*_s_)
18:2_18:2(*sn*-2)_18:1	44	54	5		20.9	no separation
18:1_18:1(*sn*-2)_18:3	44	54	5		22.1	no separation
18:1_18:1(*sn*-2)_18:2	46	54	4		21.4	no separation
16:0_16:0_18:2	46	50	2	18:2/16:0/16:0	24.8	
				16:0/16:0/18:2	25.7	1.10
				16:0/18:2/16:0	27.3	1.87
16:0_18:1_18:1	48	52	2	18:1/16:0/18:1	24.8	
				18:1/18:1/16:0	25.8	1.11
				16:0/18:1/18:1	27	1.46
16:0_16:0_18:1	48	50	1	18:1/16:0/16:0	27.2	
				16:0/16:0/18:1	28.8	1.51
				16:0/18:1/16:0	30.4	1.64
16:0_18:2_18:2	44	52	4	18:2/16:0/18:2	^a^44.9	
				18:2/18:2/16:0	46.4	1.53
				16:0/18:2/18:2	46.9	0.44
18:0_18:1_18:1	50	54	2	18:1/18:0/18:1	^a^47.4	
				18:1/18:1/18:0^b^	49.7	1.93
				18:0/18:1/18:1^b^	52.2	2.21

a Mobile phase gradient differed from that used in the analysis of other VOs.

b Enantiomeric elution order tentatively determined

Eight asymmetric TG species were analyzed in this study, and five of them, containing both SFA (C16:0 or C18:0) and UFA (C18:1 or C18:2) at the *sn*-1 and *sn*-3 positions, were simultaneously separated into enantiomers and regioisomers using the optimized chromatographic conditions (Figure 2). TGs that contained only UFAs were not separated into isomers. This is likely due to the inability to differentiate UFAs at the *sn*-1 and *sn*-3 positions of TGs by the experimented column-solvent combination.^26^ As expected, the isomeric elution profile of TG 16:0_18:1_18:1 was very similar to that reported by Masuda et al.^35^ In that study, the separation of TG 16:0_18:1_18:1 isomers was achieved within 40 min, which was slightly longer than the time needed in the present study (24–28 min). Several methodological differences discussed in the chapter Method Adaptation and Evaluation may account for the variation in retention times between the current and previous studies, including column length, modifier gradient, and back pressure.

**Figure 2:**
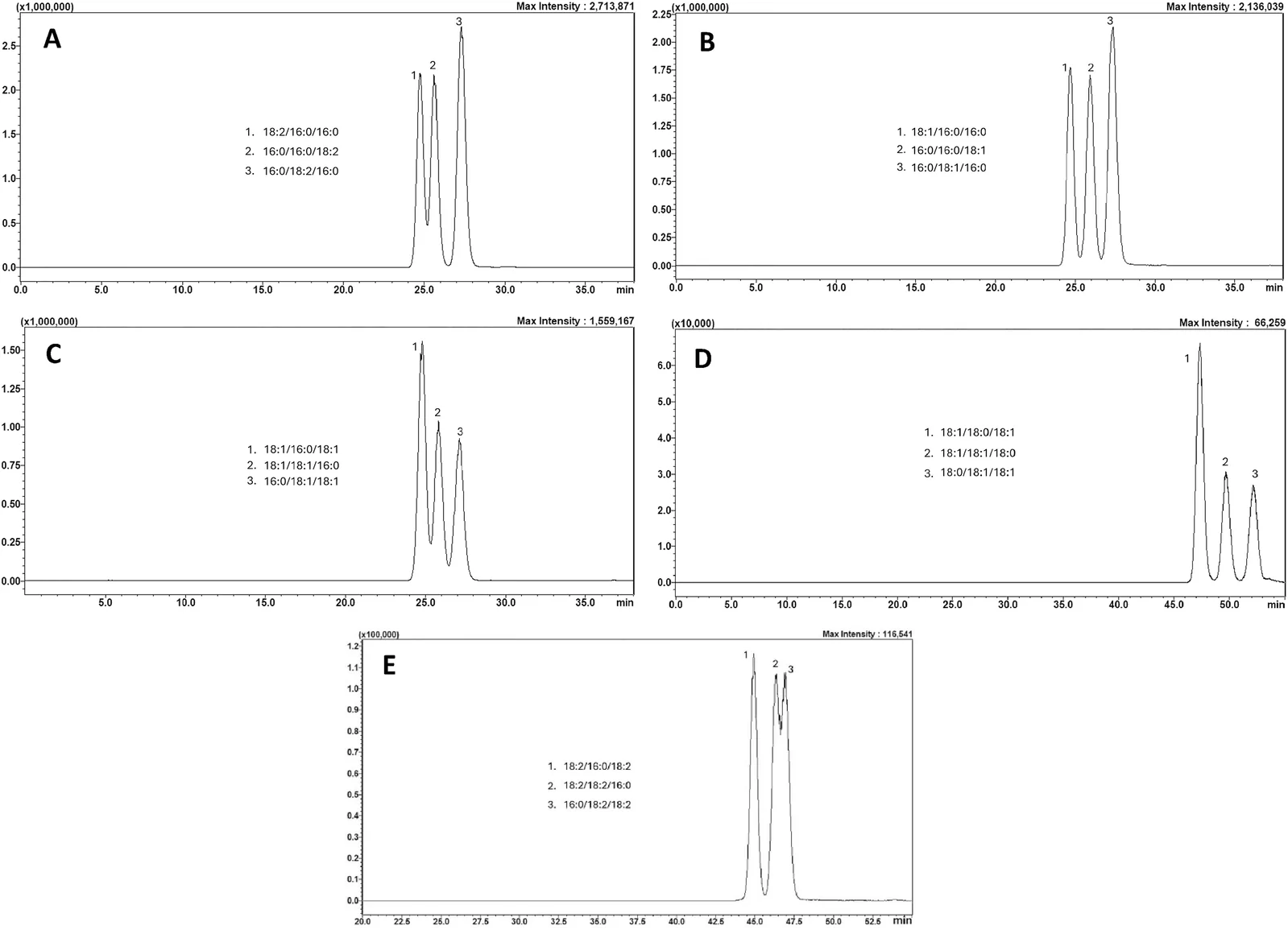
MRM chromatograms showing isomeric separation of (A) TG 16:0_16:0_18:2 at *m*/*z* 848.80 > 551.50; (B) TG 16:0_16:0_18:1 at *m*/*z* 850.80 > 551.50; (C) TG 16:0_18:1_18:1 at *m*/*z* 876.80 > 603.50; (D)*^¤^ TG 18:0_18:1_18:1 at *m*/*z* 904.80 > 603.50 and (E)* 16:0_18:2_18:2 at *m/z* 872.80 > 599.50. *Mobile phase gradient differed from other analyses. ^¤^Enantiomeric elution order tentatively determined.

Previously, Nagai et al.^26^ reported simultaneous separation of regioisomers and enantiomers of the same TG molecular species using chiral-phase HPLC coupled with MS. They employed the CHIRALPAK IF-3 [4.6 mm × 250 mm × 3 μm; amylose tris (3-chloro-4-methylphenyl carbamate) immobilized on silica gel] column with acetonitrile as a mobile phase to achieve simultaneous separation of regio- and enantiomers. The profiles of chromatograms were very similar to those obtained in the present study using chiral-phase SFC separation. However, the resolution of the separated isomers was not reported in the study by Nagai et al.^26^ Moreover, Lísa and Holčapek (2013)^19^ were unable to separate the isomers of TG 16:0_18:2_18:2 and 16:0_16:0_18:2 using a chiral-phase HPLC method with a Lux Cellulose-1 column and the mobile phase consisting of hexane and hexane-2-propanol, whereas in the present study, the isomers of these TG species were successfully resolved.

For TGs containing two SFAs and one UFA (SSU-type TGs), the retention time order of the isomers was USS < SSU < SUS (Figures S1, S2, Figure 2A,B), and for SUU-type TGs the retention time order of the isomers was USU < UUS < SUU (Figures S3, S4, Figure 2C,E). The elution order of the enantiomers of TG 18:0_18:1_18:1 (Figure S5, Figure 2D) was assigned based primarily on the consistent elution behavior observed for the other two SUU-type TG standards analyzed in this study and previously reported under comparable chiral chromatographic conditions.^35,39^ In TG 16:0_18:1_18:1 and 16:0_18:2_18:2, enantiomer with the UFA in the position *sn*-1 eluted faster than the corresponding enantiomer with the UFA in the position *sn*-3. Accordingly, TG 18:1_18:1_18:0 was assigned to elute before TG 18:0_18:1_18:1. Similar positional effects were observed previously using chiral-phase HPLC with methanol as the mobile phase^25,26^ providing additional support for the assignment. In addition, we observed that as the carbon number of SFA increased (C16 vs C18), enantiomeric separation occurred more slowly (both the retention time and the time needed for peak separation were longer, Figure 2C,D) and *R*_s_ was higher (Table 3). Altogether, the isomers that were baseline separated (*R*_s_ ≥ 1.5, Table 3) included TG 16:0/16:0/18:2 and 16:0/18:2/16:0 (Figure 2A), TG 18:1/18:1/16:0 and 16:0/18:1/18:1 (Figure 2C) and all the isomers of TG 16:0_16:0_18:1 (Figure 2B) and 18:0_18:1_18:1 (Figure 2D).

The results obtained suggest that ECN, acyl carbon number (ACN), or DB number can provide useful indications of retention behavior. However, additional structural factors such as intermolecular interactions and steric hindrances influence chiral separation.^46^ Therefore, the elution order of TG isomers cannot be assigned unequivocally using these parameters alone. Accurate isomer identification requires consideration of characteristic chromatographic behavior, comparison with published retention patterns obtained using identical chiral chromatographic conditions, and, whenever available, analysis of enantiostructured reference compounds.

### Relative Abundance of Triacylglycerol Isomers in Selected Vegetable Oils

Five distinct TG molecular species in 12 VOs were selected for stereospecific separation based on the TG composition that was recently published by our group.^17^ Three of the TGs were SUU-type ( 16:0_18:1_18:1, 18:0_18:1_18:1, and 16:0_18:2_18:2), while two were SSU-type (16:0_16:0_18:1, and 16:0_16:0_18:2). For TG 16:0_16:0_18:1 and 16:0_18:1_18:1, correction for M+2 isotopic contributions from the corresponding more unsaturated TG species was applied during data processing. A detailed description of the correction procedure is provided in the Supporting Information.

To compare the structural differences and positional preferences of SFAs and UFAs within different TG molecular species, TGs were divided into two groups: TGs with one SFA and two UFAs (SUU) and TGs with two SFAs and one UFA (SSU). To illustrate the differences, the relative area proportions (%) of TG isomers within the SUU-type TGs are presented in Figure 3A–C, and those of SSU-type TGs in Figures 4A,B. The VOs analyzed could be grouped into four categories: fruit pulp oils (avocado, olive, palm), nut oils (macadamia, peanut, walnut), cereal-derived oils (corn, oat, rice bran), and seed oils (rapeseed, sesame, sunflower). The complete data set, including the relative abundance of isomers and standard deviations, is provided in Supporting Table S3. Analysis of all TGs revealed clear positional preferences rather than random distribution of FAs and 1:1:1 abundance ratio among the isomers, and the relative proportions of the three possible stereospecific isomers differed systematically among the VOs studied. Overall, SFAs showed a strong preference for the primary positions (*sn*-1/3), while UFAs favored the *sn*-2 position.

**Figure 3:**
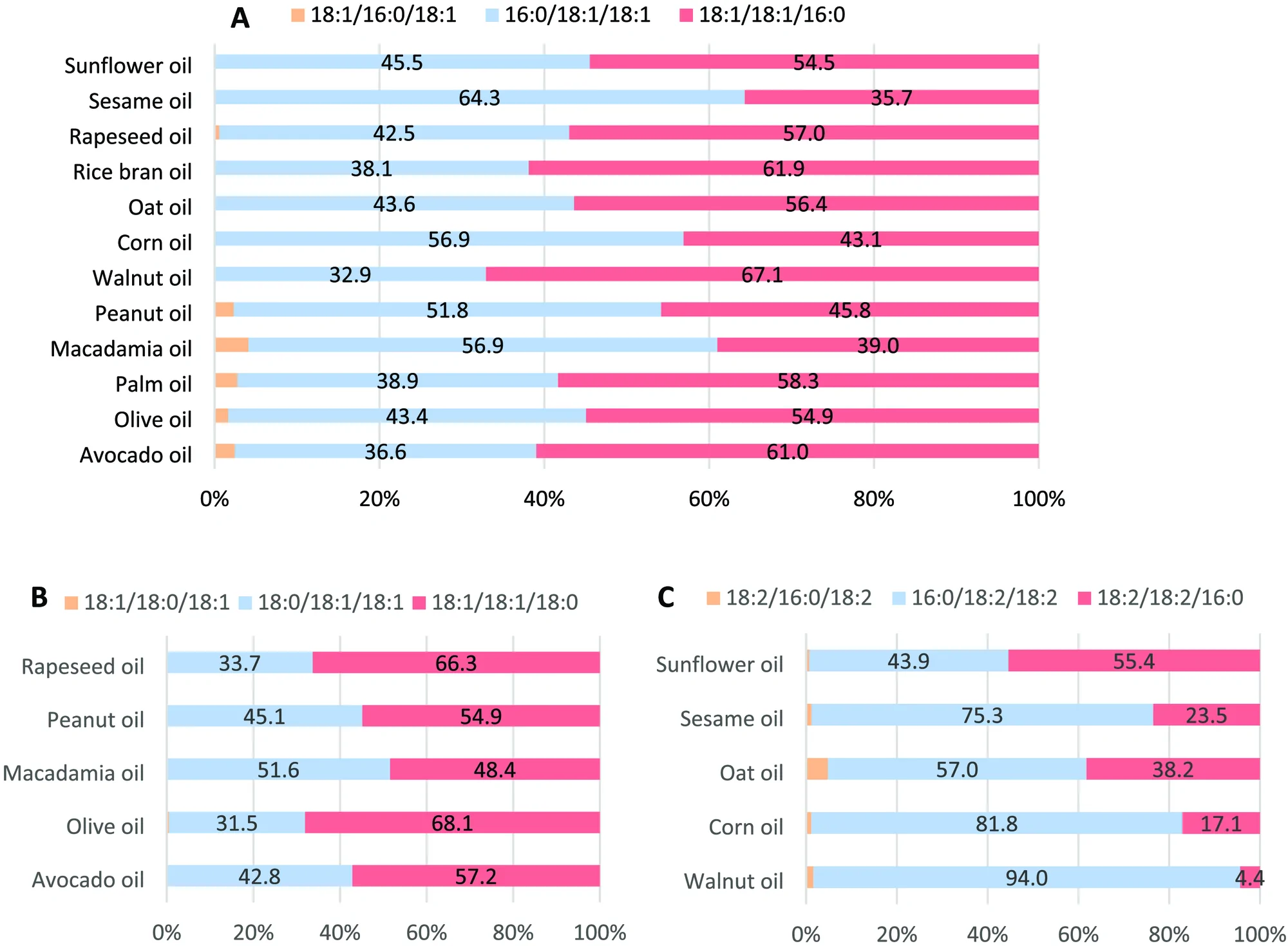
Area percentages of TG isomers in SUU-type TGs, A. 16:0_18:1_18:1, B^¤^. 18:0_18:1_18:1 and C. 16:0_18:2_18:2. The abundance of the symmetrical USU-type isomers is below 5% in all oils studied and is therefore not clearly visible in the bar chart. Detailed data set (relative abundance of isomers and standard deviations) is provided in Supporting Table S3. ^¤^Enantiomers tentatively assigned.

**Figure 4:**
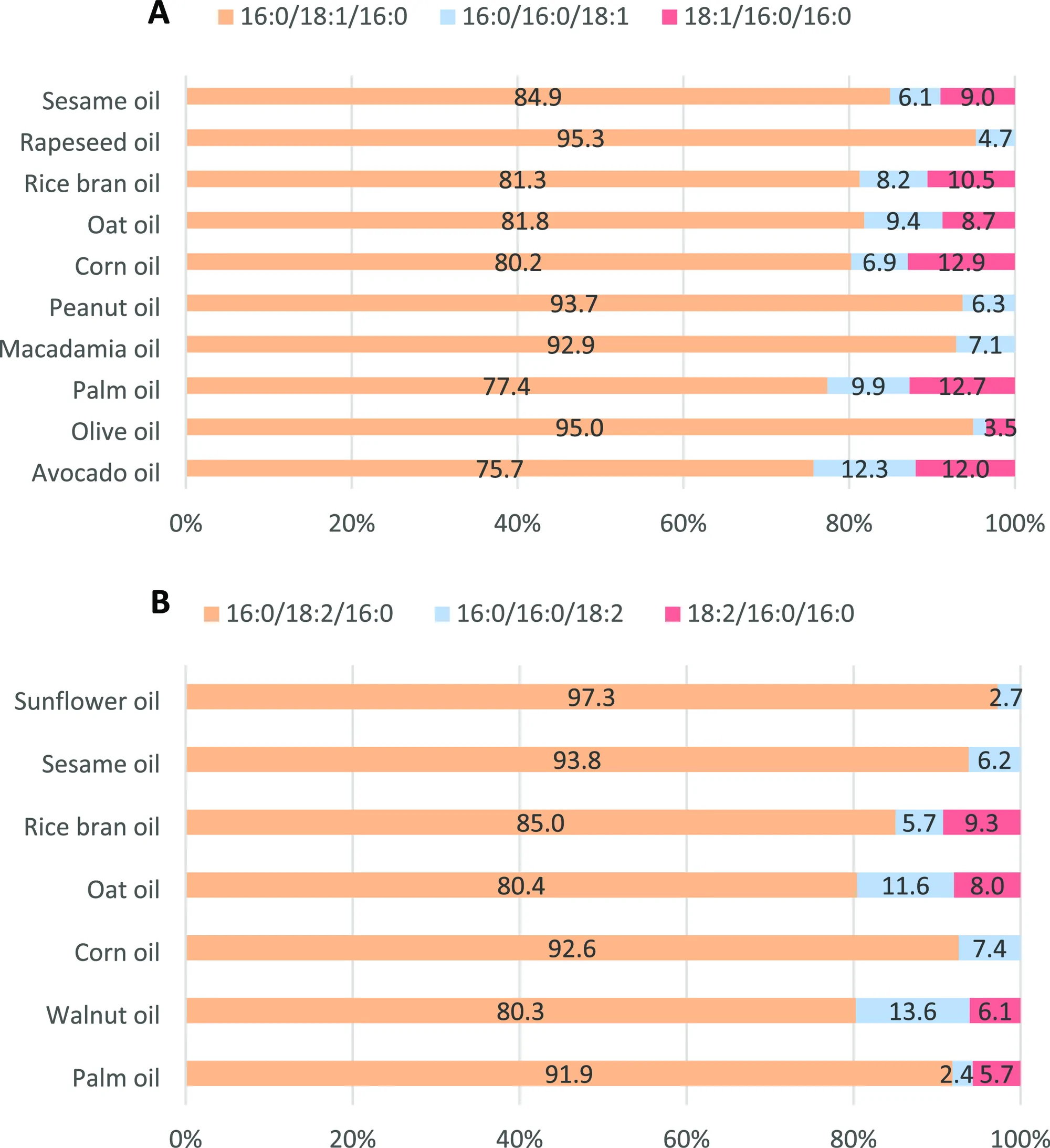
Area percentages of TG isomers in SSU-type TGs, A. 16:0_16:0_18:1 and B. 16:0_16:0_18:2.

### SUU-Type Triacylglycerols

In SUU-type TGs (Figure 3) the U/S/U isomer was consistently a minor component in all VOs, with an average abundance of approximately 2%. The only notable exception was TG 18:2/16:0/18:2 in oat oil, which has an abundance of 4.8% (Figure 3C, Table S3). The highest U/S/U proportions otherwise occurred in fruit pulp oils (olive, avocado, and palm oils at 2–3%) and in nut oils (peanut and macadamia oils at 2–4%), reflecting their distinctive TG assembly patterns.

Across SUU-type TG molecular species, the relative abundance of isomers depended on the type of UFA present in the molecule. When the UFA was C18:2, the S/U/U enantiomer showed higher abundance than U/U/S in four of the five VOs studied (Figure 3C). In contrast, when the UFA was C18:1, the U/U/S enantiomer tended to dominate over S/U/U in most of the VOs studied (Figure 3A,B).

For C18:1 rich SUU-type TGs, the SFA was predominantly esterified at *sn*-3 position. In TG 16:0_18:1_18:1, eight out of 12 oils predominantly formed the U/U/S isomer, and a similar trend was observed in TG 18:0_18:1_18:1, where four out of five oils favored *sn*-3 acylation of the SFA. Across most oils, the average U/U/S:S/U/U ratio was 60:40, confirming an overall *sn*-3 preference for SFA.

The highest proportions of enantiomer 18:1/18:1/16:0 (58–67%) were found in walnut, rice bran, avocado, and palm oils, whereas rapeseed, oat, olive, and sunflower oils showed moderately lower proportions (54–57%). In contrast, the U/U/S:S/U/U ratio of C18:1 rich enantiomers was reversed in macadamia oil (40:57 in 16:0_18:1_18:1 and 48:52 in 18:0_18:1_18:1), and a similar pattern was also observed for TG 16:0_18:1_18:1 in peanut, corn, and sesame oils (U/U/S:S/U/U ratio 42:58 on average). These oils showed lower proportions of the U/U/S isomer (36–48%) (Figure 3A–B).

The strong *sn*-3 preference for SFAs in C18:1 rich SUU-type TGs (TG 16:0_18:1_18:1 and 18:0_18:1_18:1) reflects enzymatic selectivity during TG biosynthesis, likely driven by steric and kinetic factors (Cai et al., 2025). However, the C18:2 rich SUU-type TG 16:0_18:2_18:2 deviated markedly from this pattern. Only one out of five oils (sunflower oil) favored *sn*-3 esterification of C16:0, while walnut, corn, sesame, and oat oils predominantly esterified C16:0 at *sn*-1 position, forming the 16:0/18:2/18:2 enantiomer at high proportions (94, 82, 75, and 57%, respectively). These results indicate that the positional behavior of saturated acyl chains differs fundamentally between C18:1 and C18:2 rich SUU-type TGs.

Only a few studies have reported complete isomeric ratios for SUU-type TGs, but the values observed here were broadly consistent with earlier findings. For palm oil, the corrected ratio of the three SUU-type isomers (18:1/16:0/18:1, 16:0/18:1/18:1, and 18:1/18:1/16:0) was 3:39:58, closely matching the ratios reported earlier by Nagai et al. (7:39:54)^27^ and Masuda et al. (9:33:58).^35^ Minor differences between studies may result from isotopic overlap in MS-based analyses, data processing approaches, or natural genetic and environmental variability among samples. Masuda et al. (2021) also reported regioisomeric ratios of TG 16:0_18:1_18:1 in refined and extra virgin olive oil (7:41:52 and 6:39:55, respectively), and our value for commercial olive oil (2:43:55) was in close agreement. The positional distribution of FAs in olive oil is known to be strongly influenced by cultivar and genetic background.^18^

### SSU-Type Triacylglycerols

Clear positional preferences of UFA for *sn*-2 position were observed, reflecting differences in molecular symmetry between SUU- (Figure 3) and SSU-(Figure 4) type TGs. In SUU-type TGs, FAs were predominantly arranged as asymmetric TGs (S/U/U or U/U/S), whereas SSU-type TGs were dominated by symmetric S/U/S isomers. The highest proportion of S/U/S was found in sunflower oil (97% 16:0/18:2/16:0), followed by olive oil (95% 16:0/18:1/16:0). The strong enrichment of UFAs at *sn*-2 position is consistent with earlier findings^47^ and the well-known enzymatic preferences of LPAAT and DGAT.^11^ Across all TGs, C18:1 and C18:2 strongly favored the *sn*-2 position, whereas isomers with C16:0 or C18:0 at *sn*-2 represented much lower proportion (average abundance of U/S/U, S/S/U, and U/S/S 4.4%).

Unlike SUU-type TGs, where C18:1 and C18:2 rich species differed distinctly, SSU-type TGs showed much weaker differences between molecules containing C18:1 or C18:2 acids (Figure 4). The only minor trend distinguishing the SSU species was the slightly higher abundance of U/S/S isomer (SFA at *sn*-3) in C18:1 containing TGs. For TG 16:0_16:0_18:1, the U/S/S enantiomer (18:1/16:0/16:0) accounted for an average of 7% of the isomer distribution, and five of ten oils (50%) predominantly formed this enantiomer. In contrast, the analogous C18:2 containing TG 16:0_16:0_18:2 showed a lower average proportion of 4% for the corresponding enantiomer (18:2/16:0/16:0), with only two of seven oils (29%) favoring this enantiomeric configuration. Furthermore, the relative abundance of 16:0/18:2/16:0 (89%) was slightly higher than that of 16:0/18:1/16:0 (86%) within respective TG molecular species, indicating a stronger preference to *sn*-2 position when the DB number increased in FA. Overall, these results demonstrate that SSU-type TG species are dominated by S/U/S isomer due to the strong *sn*-2 selectivity for UFAs, with only minor differences observed between C18:1 and C18:2 containing TGs.

The SSU-type TG data also agreed with the previously reported regioisomeric distributions. Kallio et al. reported that TG 16:0_16:0_18:1 in palm oil occurred in a regioisomeric ratio of 22:78 between 16:0_16:0­(*sn-*2)_18:1 and 16:0_18:1­(*sn*-2)_16:0,^16^ which closely matched the ratio observed in this study (23:77). Similarly, the SSU-type TG isomer patterns observed in olive oil, characterized by high proportions of 16:0/18:1/16:0 and 16:0/18:2/16:0 isomers, were consistent with earlier work showing strong *sn*-2 selectivity for UFAs in this species.^18,35^ Overall, the SSU-type TG results confirm that the *sn*-2 enrichment of C18:1 and C18:2 is highly conserved across VOs and largely independent of species.

### Biological Relevance and Need for Future Research

The difference in TG isomeric profiles among VOs reflects interspecies variation in key steps of lipid biosynthesis pathways in plants. The results of this study confirmed that the stereospecific distribution of FAs in VO TGs is highly structured and species-specific, rather than being random. Clear differences were observed in SUU-type TGs, particularly between C18:1 and C18:2 rich molecular species. The contrasting positional behavior between C18:1 and C18:2 rich SUU-type TGs may have arisen from differences in acyl-CoA pool composition and the degree of unsaturation, both of which influence acyltransferase substrate selectivity and *sn*-positional preferences.^11,12^

TGs are synthesized through several metabolic pathways, the most predominant being the acyl-CoA-dependent Kennedy pathway. In this pathway, GPAT acylates *sn*-1 of glycerol-3-phosphate, LPAAT incorporates an acyl chain to *sn*-2 with a strong preference for unsaturated acyl-CoAs, and DGAT completes TG biosynthesis by esterifying the *sn*-3 position. These enzymatic preferences collectively explain the characteristic SUU- and SSU-type patterns observed across the examined VOs.

Isomeric structures of TGs play a key role in determining the nutritional and functional properties of edible oils. The presence of UFAs at the *sn*-2 position enhances intestinal absorption and bioavailability of UFAs, which in turn may contribute to more favorable lipid metabolic responses,^48,49^ whereas SFAs at *sn*-1 and *sn*-3 are hydrolyzed earlier and absorbed mainly as free fatty acids. Positional isomerism also affects crystallization and melting behavior, influencing textural properties important in spreads and confectionery fats, as well as oxidative stability.^8,50–52^ Symmetric SUS-type TGs (e.g., 16:0/18:1/16:0) tend to pack more efficiently because they lack the steric disruption caused by a kinked UFA in positions *sn*-1 or *sn*-3, which increases steric hindrance and reduces the ability of TG molecules to pack tightly.^53^ As a result, symmetric TGs form more stable crystals, thereby enhancing plasticity and structural stability in fat-based products. In contrast, oils enriched in C18:2 containing SUU-type TGs (e.g., 16:0_18:2_18:2) are more susceptible to oxidation, often requiring the incorporation of antioxidants to ensure product shelf life, whereas symmetric SUS-type TGs (e.g., 16:0_18:1_16:0) contribute to improved oxidative stability alongside their crystallization advantages. The ability to resolve TG enantiomers and regioisomers in a single analysis provides new opportunities to link lipid structure with biological function and technological performance. Understanding interspecies differences in TG structures will also support the authenticity assessment, product formulation, and the design of products with targeted physical or nutritional properties.

To our knowledge, this is the first time that chiral SFC-MS has been used to characterize the regioisomeric and enantiomeric distributions of five TG molecular species across a broad range of VOs. This approach can also be applied to study more complex lipid matrices, such as human milk, for which detailed TG structural information is becoming increasingly important in terms of nutrition, physiology, and biochemistry.

However, using the current method, enantiomeric separation was unsuccessful for TGs containing two UFAs at *sn*-1 and *sn*-3 positions. Further development of the analytical methods using alternative stationary phases, mobile phases, or other methodological modifications is necessary to enhance the chromatographic separation of structurally similar TGs, particularly those TGs composed only of SFAs or UFAs. Future studies are also needed to quantify the absolute concentrations of TG isomers in VOs, as well as to determine the impact of cultivars, varieties, and growing environments on the content and composition of TG isomers.

## Supplementary Material



## Notes

Tables listing TG reference compounds, analytical conditions tested, and percentages of TG isomers; identification strategy for TG isomers, elution gradient program, and description of TG peak area correction for the chromatographically overlapping M+2 isotopes (PDF)
