# Supplementary material for: Stereospecific Characterization of Triacylglycerols in Vegetable Oils by Chiral Supercritical Fluid Chromatography and Mass Spectrometry
Source: J Agric Food Chem. 2026 Oct 24;74(43):34221–32. doi: 10.1021/acs.jafc.6c07006 (PMC13741822; doi:10.1021/acs.jafc.6c07006)
Supplement: Supplementary file 1 [file jf6c07006_si_001.pdf]

Stereospecific Characterization of Triacylglycerols in Vegetable Oils by Chiral Supercritical Fluid Chromatography and Mass Spectrometry

Marika KALPIO[a&], Dilakshi NAOTUNNA PALLIYA GURUGE[a&], Mikael FABRITIUS[a], Qizhu ZHAO[a], Baoru YANG[a]*

[a]Food Sciences Unit, Department of Life Technologies, University of Turku, FI-20014 Turku, Finland

[&]These authors contributed equally to the manuscript.

Supporting Information

**Page**	**Contents**
S2	**Table S1.** Triacylglycerol reference compounds.
S3	**Table S2.** Analytical conditions tested.
S4	**Table S3.** Percentages of triacylglycerol isomers.
S5	**Identification Strategy** for Triacylglycerol Isomers, **Figures S1−S6**.
S8	**Figure S7.** Elution gradient programme of the method optimized in the current study (A) and the method published by Masuda et al. 2021 (B).
S9	**Description** of Triacylglycerol Peak Area Correction for the Chromatographically Overlapping M+2 Isotopes

Table S1. Triacylglycerol reference compounds.

Full name	Abbreviation
**1,3-dioleoyl-2-palmitoyl-*sn*-glycerol**	18:1_16:0(*sn*-2)_18:1
**1,3-dipalmitoyl-2-oleoyl-*sn*-glycerol**	16:0_18:1(*sn*-2)_16:0
**1,3-dioleoyl-2-stearoyl-*sn*-glycerol**	18:1_18:0(*sn*-2)_18:1
**1,3-dilinoleoyl-2-palmitoyl-*sn*-glycerol**	18:2_16:0(*sn*-2)_18:2
**1,3-dipalmitoyl-2-linoleoyl-*sn*-glycerol**	16:0_18:2(*sn*-2)_16:0
**1,3-dilinoleoyl-2-oleoyl-*sn*-glycerol**	18:2_18:1(*sn*-2)_18:2
**1,3-dioleoyl-2-linolenoyl-*sn*-glycerol**	18:1_18:3(*sn*-2)_18:1
**1,3-dioleoyl-2-linoleoyl-*sn*-glycerol**	18:1_18:2(*sn*-2)_18:1
**1,2-dioleoyl-3-palmitoyl-*sn*-glycerol**	18:1/18:1/16:0*
**1-oleoyl-2,3-dipalmitoyl-*sn*-glycerol**	18:1/16:0/16:0*
**1,2-dilinoleoyl-3-palmitoyl-*sn*-glycerol**	18:2/18:2/16:0*
**1-linoleoyl-2,3-dipalmitoyl-*sn*-glycerol**	18:2/16:0/16:0*
**1,2(2,3)-dioleoyl-3(1)-palmitoyl-*rac*-glycerol**	18:1_18:1(*sn*-2)_16:0
**1,2(2,3)-dipalmitoyl-3(1)-oleoyl-*rac*-glycerol**	18:1_16:0(*sn*-2)_16:0
**1,2(2,3)-dioleoyl-3(1)-stearoyl-*rac*-glycerol**	18:1_18:1(*sn*-2)_18:0
**1,2(2,3)-dilinoleoyl-3(1)-palmitoyl-*rac*-glycerol**	18:2_18:2(*sn*-2)_16:0
**1,2(2,3)-dipalmitoyl-3(1)-linoleoyl-*rac*-glycerol**	16:0_16:0(*sn*-2)_18:2
**1,2(2,3)-dilinoleoyl-3(1)-oleoyl-*rac*-glycerol**	18:2_18:2(*sn*-2)_18:1
**1,2(2,3)-dioleoyl-3(1)-linolenoyl-*rac*-glycerol**	18:1_18:1(*sn*-2)_18:3
**1,2(2,3)-dioleoyl-3(1)-linoleoyl-*rac*-glycerol**	18:1_18:1(*sn*-2)_18:2

*Asymmetric enantiostructured TG reference compounds synthesized by the University of Iceland.

Table S2. Analytical conditions tested.

Method number	Back pressure (MPa)	Column oven temperature (^{o}C)	Flow rate (mL/min)	Modifier gradient	Scan range (*m/z*)	Injection volume (μL)
**01**	10	25	0.6	5–35% (40 min), 35–5% (0.01 min), and 5% (5 min)	800–1000	1
**02**	12	25	0.6	5–35% (40 min), 35–5% (0.01 min), and 5% (5 min)	800–1000	1
**03**	16	25	0.6	5–35% (40 min), 35–5% (0.01 min), and 5% (5 min)	800–1000	1
**04**	10	25	0.8	5–35% (40 min), 35–5% (0.01 min), and 5% (5 min)	800–1000	1
**05**	12	25	0.8	5–35% (40 min), 35–5% (0.01 min), and 5% (5 min)	800–1000	1
**06**	16	25	0.8	5–35% (40 min), 35–5% (0.01 min), and 5% (5 min)	800–1000	1
**07**	10	30	0.6	5–35% (40 min), 35–5% (0.01 min), and 5% (5 min)	800–1000	1
**08**	10	25	0.4	5–35% (40 min), 35–5% (0.01 min), and 5% (5 min)	800–1000	1
**09**	10	25	0.4	5–35% (40 min), 35–5% (0.01 min), and 5% (5 min)	800–1000	0.5
**10**	10	35	0.6	5–35% (40 min), 35–5% (0.01 min), and 5% (5 min)	800–1000	0.5
**11**	12	25	0.4	5–35% (40 min), 35–5% (0.01 min), and 5% (5 min)	800–1000	0.5
**12**	12	30	0.4	5–35% (40 min), 35–5% (0.01 min), and 5% (5 min)	750–950	0.5
**13**	12	25	0.4	5–35% (10 min), 35–40% (35 min), 40–5% (0.01 min), and 5% (5 min)	750–950	0.5
**14**	12	25	0.4	Isocratic 35% (40 min)	750–950	0.5
**15**	12	25	0.4	5–35% (10 min), 35–40% (25 min), 40–5% (0.01 min), and 5% (3 min)	750–950	0.5

Table S3. Area percentages of isomers within each triacylglycerol molecular species.

	Fruit pulp oils						Nut oils						Cereal-derived oils						Seed oils					
sn-TG	Avocado oil		Olive oil		Palm oil		Macadamia oil		Peanut oil		Walnut oil		Corn oil		Oat oil		Rice bran oil		Rapeseed oil		Sesame oil		Sunflower oil	
	%	STD	%	STD	%	STD	%	STD	%	STD	%	STD	%	STD	%	STD	%	STD	%	STD	%	STD	%	STD
OPO	2.4	0.2	1.7	0.4	2.7	0.1	4.1	0.2	2.3	0.1	0.0	0.0	0.0	0.0	0.0	0.0	0.0	0.0	0.6	0.1	0.0	0.0	0.0	0.0
POO	36.6	0.4	43.4	1.1	38.9	1.0	56.9	0.4	51.8	1.0	32.9	0.5	56.9	1.9	43.6	1.3	38.1	1.0	42.5	0.7	64.3	0.2	45.5	1.0
OOP	61.0	0.6	54.9	1.5	58.3	1.2	39.0	0.2	45.8	1.1	67.1	0.5	43.1	1.9	56.4	1.3	61.9	1.0	57.0	0.6	35.7	0.2	54.5	1.0
OSO	n.d.		0.5	0.7	n.a.		n.d.		n.d.		n.a.		n.a.		n.a.		n.a.		n.d.		n.a.		n.a.	
SOO*	42.8	0.0	31.5	1.2	n.a.		51.6	0.4	45.1	1.4	n.a.		n.a.		n.a.		n.a.		33.7	0.8	n.a.		n.a.	
OOS*	57.2	0.0	68.1	1.8	n.a.		48.4	0.4	54.9	1.4	n.a.		n.a.		n.a.		n.a.		66.3	0.8	n.a.		n.a.	
LPL	n.a.		n.a.		n.a.		n.a.		n.a.		1.6	0.5	1.2	0.1	4.8	0.1	n.a.		n.a.		1.2	0.0	0.7	0.1
PLL	n.a.		n.a.		n.a.		n.a.		n.a.		94.0	1.5	81.8	0.3	57.0	3.2	n.a.		n.a.		75.3	0.1	43.9	1.1
LLP	n.a.		n.a.		n.a.		n.a.		n.a.		4.4	1.0	17.1	0.2	38.2	3.3	n.a.		n.a.		23.5	0.1	55.4	1.2
POP	75.7	1.4	95.0	0.1	77.4	0.1	92.9	0.1	93.7	0.4	n.a.		80.2	0.8	81.8	2.2	81.3	0.7	95.3	2.3	84.9	2.5	n.a.	
PPO	12.3	1.3	1.6	0.0	9.9	0.7	7.1	0.1	6.3	0.4	n.a.		6.9	0.7	9.4	1.9	8.2	0.1	4.7	2.3	6.1	2.4	n.a.	
OPP	12.0	0.1	3.5	0.1	12.7	0.6	0.0	0.0	0.0	0.0	n.a.		12.9	0.0	8.7	0.3	10.5	0.7	0.0	0.0	9.0	0.2	n.a.	
PLP	n.a.		n.a.		91.9	0.1	n.a.		n.a.		80.3	0.1	92.6	0.0	80.4	0.9	85.0	4.3	n.a.		93.8	0.0	97.3	0.0
PPL	n.a.		n.a.		2.4	0.1	n.a.		n.a.		13.6	0.8	7.4	1.1	11.6	1.3	5.7	6.8	n.a.		6.2	0.1	2.7	0.1
LPP	n.a.		n.a.		5.7	0.0	n.a.		n.a.		6.1	0.8	0.0	1.1	8.0	0.5	9.3	2.6	n.a.		0	0.1	0	0.1

*enantiomers tentatively assigned

n.a. = not analyzed

n.d. = not detected or traces

P = C16:0

S = C18:0

O = C18:1

L = C18:2

Identification Strategy for Triacylglycerol Isomers

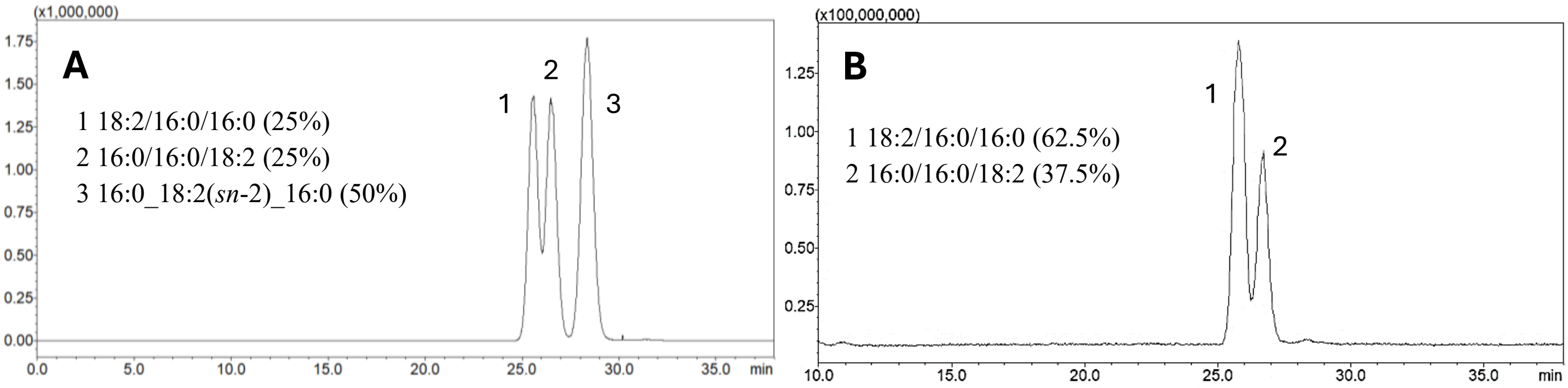

Figure S1. Chromatograms of TG 16:0_16:0(*sn*-2)_18:2 co-injected with 50% of symmetric 16:0_18:2(*sn*-2)_16:0 (A), and 16:0_16:0(*sn*-2)_18:2 co-injected with 25% of 18:2/16:0/16:0 enantiomer (B). Peak assignments and the theoretical proportions of individual isomers in the injected mixtures are indicated in the figure.

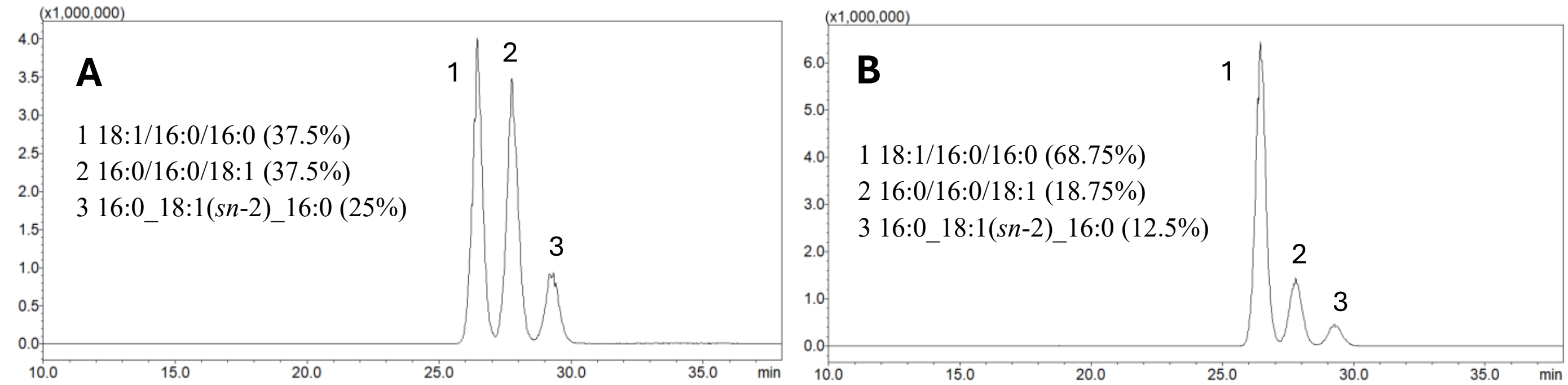

Figure S2. Chromatograms of TG 16:0_16:0(*sn*-2)_18:1 co-injected with 25% of symmetric 16:0_18:1(*sn*-2)_16:0 (A), and 16:0_16:0(*sn*-2)_18:1 co-injected with 12.5% of symmetric 16:0_18:1(*sn*-2)_16:0 and 50% of 18:1/16:0/16:0 enantiomer (B). Peak assignments and the theoretical proportions of individual isomers in the injected mixtures are indicated in the figure.

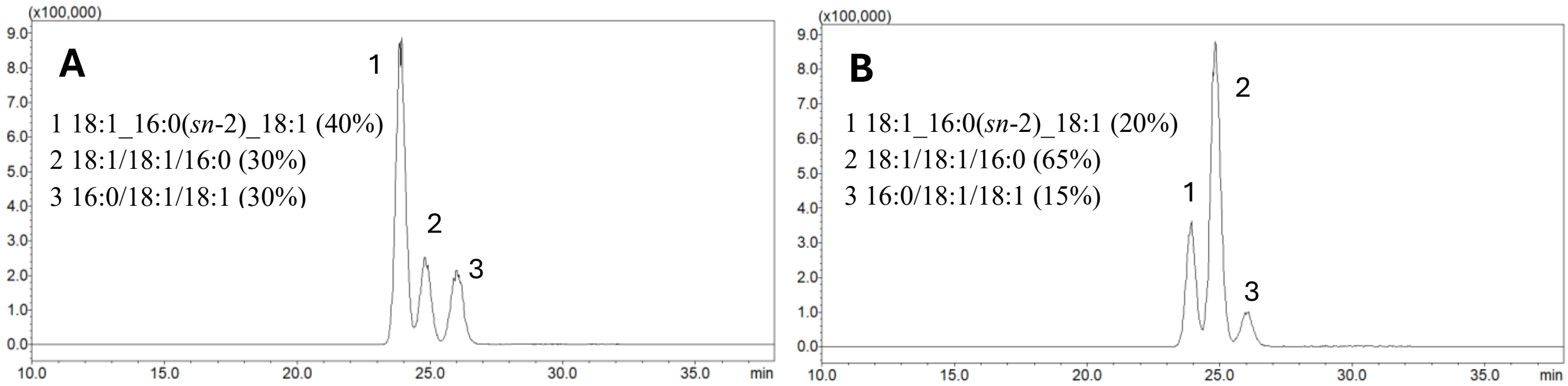

Figure S3. Chromatograms of TG 16:0_18:1(*sn*-2)_18:1 co-injected with 40% of symmetric 18:1_16:0(*sn*-2)_18:1 (A), and 16:0_18:1(*sn*-2)_18:1 co-injected with 20% of symmetric 18:1_16:0(*sn*-2)_18:1 and 50% of 18:1/18:1/16:0 enantiomer (B). Peak assignments and the theoretical proportions of individual isomers in the injected mixtures are indicated in the figure.

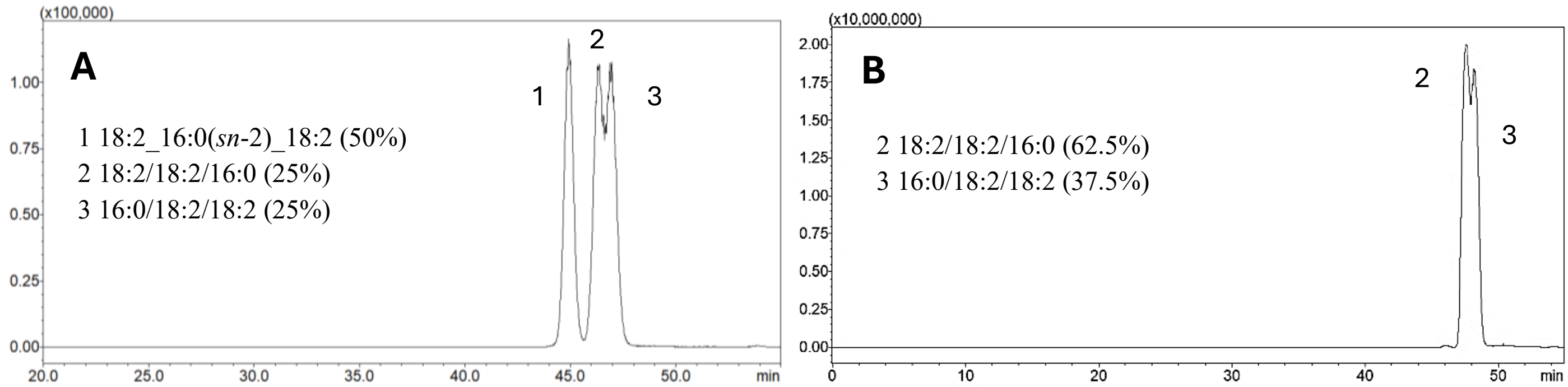

Figure S4. Chromatograms of TG 16:0_18:2(*sn*-2)_18:2 co-injected with 50% of symmetric 18:2_16:0(*sn*-2)_18:2 (A), and 16:0_18:2(*sn*-2)_18:2 co-injected with 25% 18:2/18:2/16:0 enantiomer (B). Peak assignments and the theoretical proportions of individual isomers in the injected mixtures are indicated in the figure.

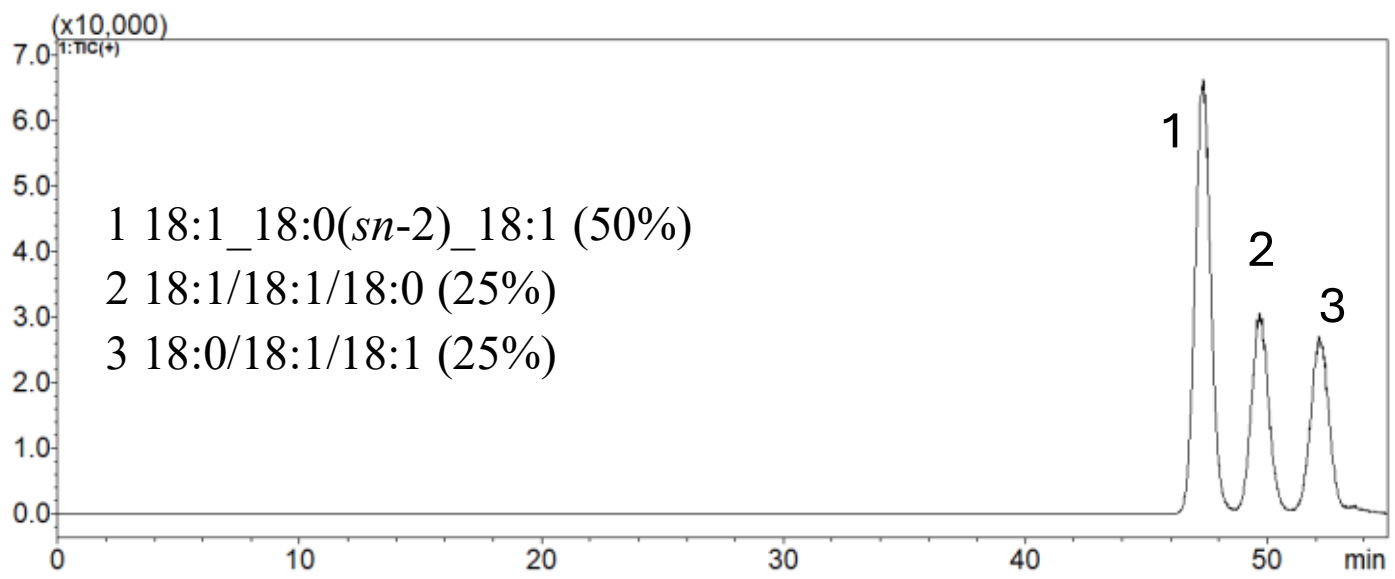

Figure S5. Chromatogram of TG 18:0_18:1(*sn*-2)_18:1 co-injected with 50% of symmetric 18:1_18:0(*sn*-2)_18:1. Peak assignments and the theoretical proportions of individual isomers in the injected mixtures are indicated in the figure.

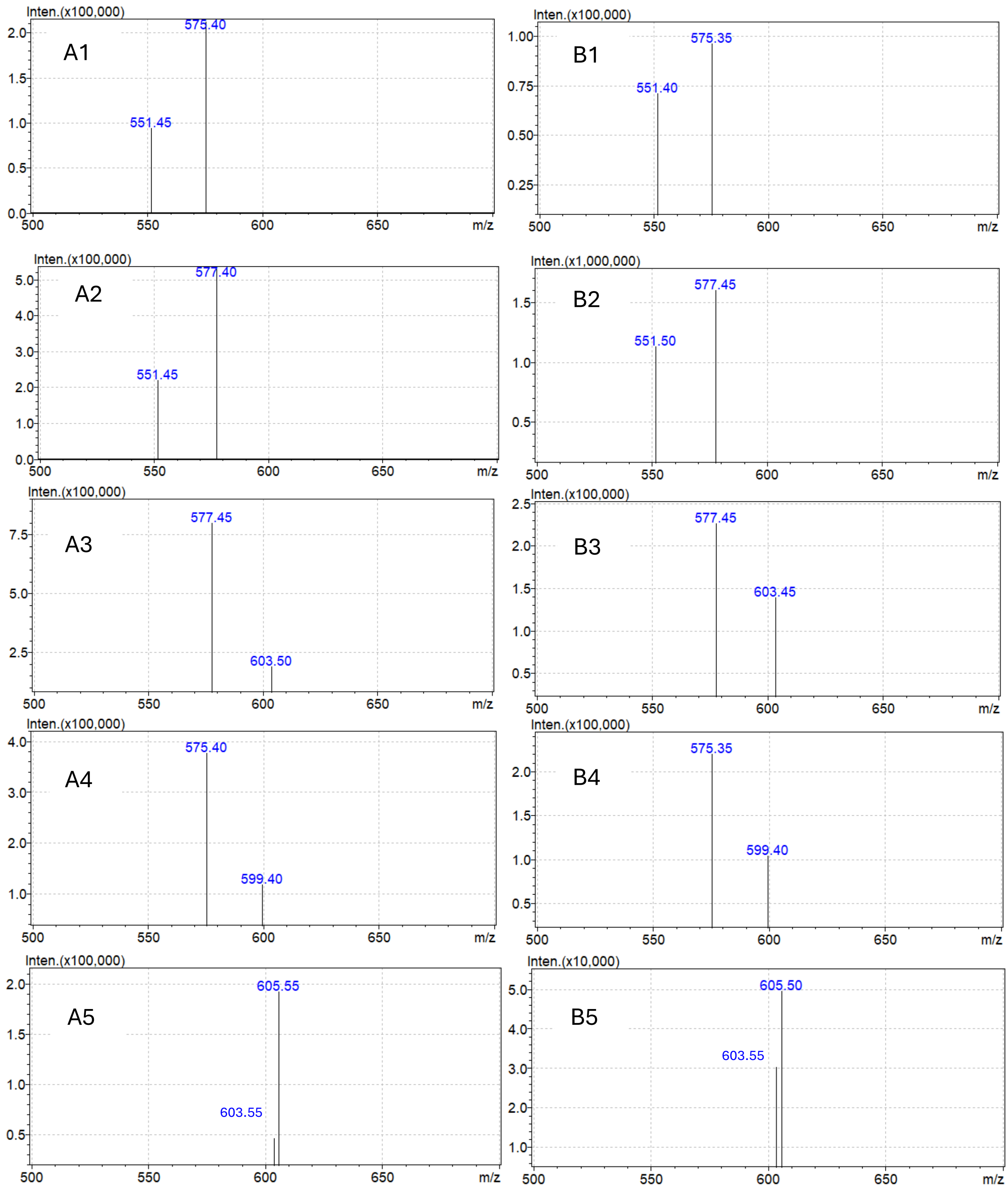

Figure S6. Product-ion spectra of symmetric (A1-A5) and asymmetric (B1-B5) TG reference compounds 16:0_16:0(*sn*-2)_18:2, 16:0_16:0(*sn*-2)_18:1, 16:0_18:1(*sn*-2)_18:1, 16:0_18:2(*sn*-2)_18:2, and 18:0_18:1(*sn*-2)_18:1, respectively.

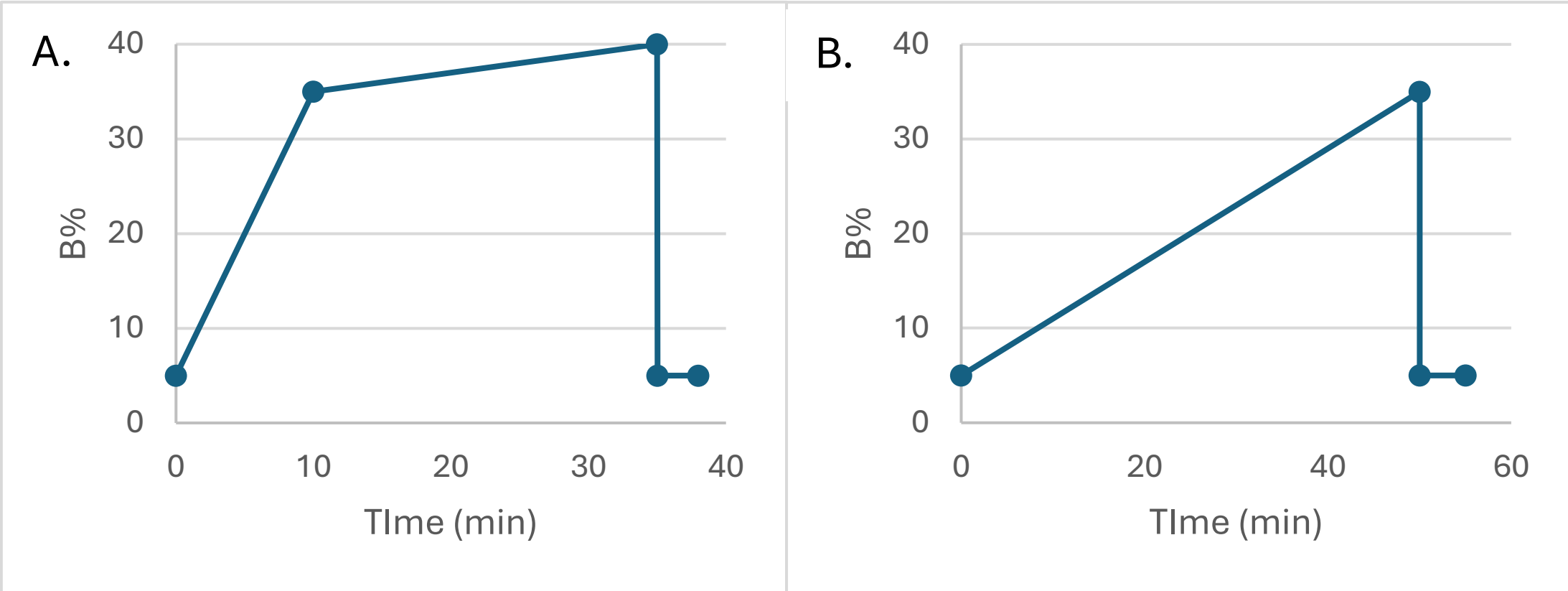

Figure S7. Elution gradient programme of the method optimized in the current study (A) and the method published by Masuda et al. 2021 (B).

Description of Triacylglycerol Peak Area Correction for the Chromatographically Overlapping M+2 Isotopes

Rationale. When quantifying a target TG, its chromatographic peak may partially overlap with the M+2 isotope peak of another TG with one more double bond. For example, in peanut oil, the $[M+NH_4]^+$ ion of TG 52:3 appears at *m/z* 874.8, and its M+2 isotope differs from the monoisotopic mass of TG 52:2 $[M+NH_4]^+$ (*m/z* 876.8) by only 0.00894 Da. Because triple-quadrupole MS operates at unit mass resolution, these small mass differences can not be resolved. Consequently, the M+2 isotope of TG 52:3 contributes to the measured signal at the nominal mass of TG 52:2, and this contribution must be subtracted from the integrated peak area to obtain accurate relative abundances. It should be noted that isotope overlap corrections are generally not required when using high-resolution mass spectrometers (e.g., Orbitrap analyzers), which are capable of resolving small mass differences between isotopic ions.

Natural Isotope Abundances. For a compound with formula $C_aH_bO_cN_d$, the monoisotopic (M+0) peak contains only the lightest isotopes (^{12}C, ^{1}H, ^{16}O, ^{14}N), while the M+1, M+2… peaks contain heavier isotopes. Neutral TGs only contain carbon, hydrogen and oxygen, but in case of ammoniated TG adducts $[M+NH_4]^+$, isotopes of nitrogen also need to be considered for maximum accuracy.

Natural isotope abundances.

Element	Isotope	Abundance (%)	Nominal mass shift
Carbon	^{12}C	98.93	0
Carbon	^{13}C	1.07	+1
Hydrogen	^{1}H	99.985	0
Hydrogen	^{2}H	0.015	+1
Oxygen	^{16}O	99.757	0
Oxygen	^{17}O	0.038	+1
Oxygen	^{18}O	0.205	+2
Nitrogen	^{14}N	99.636	0
Nitrogen	^{15}N	0.364	+1

Calculation of Isotope Distributions. Isotopic abundances were calculated by representing each element's natural isotope pattern as a probability polynomial, where the coefficients correspond to isotope abundances and the exponents represent the nominal mass shift (+0, +1, +2). The molecular isotope distribution was then obtained by polynomial multiplication of the elemental polynomials, which is mathematically equivalent to convolving the isotope probabilities of all atoms in the molecular formula.

Carbon: $P(C) = 0.9893x^0 + 0.0107x^1$

Hydrogen: $P(H) = 0.99985x^0 + 0.00015x^1$

Oxygen: $P(O) = 0.99757x^0 + 0.00038x^1 + 0.00205x^2$

Nitrogen: $P(N) = 0.99636x^0 + 0.00364x^1$

For *n* atoms of an element, the isotope pattern is the power of *n* of the base polynomial:

$$P(C_a) = [P(C)]^a$$

This is computed by repeated convolution (polynomial multiplication). For example, for 2 carbon atoms: $P(C_2) = P(C) * P(C)$

$$= [0.9893, 0.0107] * [0.9893, 0.0107]$$

$$= [0.9787, 0.0212, 0.0001]$$

Where $*$ denotes convolution. The result is an array $[P_0, P_1, P_2, \ldots]$ where:

P_0 = probability of M+0

P_1 = probability of M+1

P_2 = probability of M+2

In practice, $P(C) * P(C)$

$$= (0.9893x^0 + 0.0107x^1) \cdot (0.9893x^0 + 0.0107x^1)$$

$$= (0.9893x^0 \cdot 0.9893x^0)+(2\cdot 0.9893x^0\cdot 0.0107x^1)+(0.0107x^1\cdot 0.0107x^1)$$

$$=0.9787x^0+0.0212x^1+0.0001x^2$$

Finally, the molecular isotope pattern is the convolution of all elemental patterns:

$$\text{Isotope pattern} = P(C_a) * P(H_b) * P(O_c) * P(N_d)$$

As an example, the $[NH_4]^+$ adduct of TG 52:3 has the elemental composition of $C_{55}H_{104}O_6N$. The calculated probabilities of isotopes for the first five isotopic peaks (M+0…M+4) are shown in the table below. These five peaks account for 99.9% of the total isotope envelope.

Isotope probabilities of TG 52:3 ammonium adduct $[M+NH_4]^+$.

Isotope	Absolute probability	Relative to M+0
M+0	0.5353	1.0000
M+1	0.3298	0.6164
M+2	0.1065	0.1991
M+3	0.0239	0.0447
M+4	0.0042	0.0078

In this case, the M+2 isotope of TG 52:3 interferes with the target TG 52:2 whenever the two species are not chromatographically separated. The relative proportion of TG 52:3 M+2 isotope is multiplied by the integrated peak area of TG 52:3 within the overlap region. This calculated interference is then subtracted from the measured peak area of TG 52:2 to obtain the corrected value. If multiple TGs differing by one double bond overlap chromatographically (e.g., TG 52:4, 52:3, 52:2, 52:1), the corrections are applied sequentially, beginning with the most unsaturated TG (lowest *m/z*) and proceeding stepwise toward the most saturated species.
